# Predictive Value of Geriatric Nutritional Risk Index in Patients With Coronary Artery Disease: A Meta-Analysis

**DOI:** 10.3389/fnut.2021.736884

**Published:** 2021-09-29

**Authors:** Yu Fan, Lian He, Yongjing Zhou, Changfeng Man

**Affiliations:** Institute of Molecular Biology and Translational Medicine, The Affiliated People's Hospital, Jiangsu University, Zhenjiang, China

**Keywords:** geriatric nutritional risk index, coronary artery disease, mortality, cardiovascular events, meta-analysis

## Abstract

**Background:** Low Geriatric Nutritional Risk Index has been identified as an index of impaired nutritional state. The objective of the meta-analysis was to assess the association of the Geriatric Nutritional Risk Index (GNRI) with adverse outcomes in patients with coronary artery disease (CAD).

**Methods:** Relevant studies were identified by comprehensively searching PubMed and Embase databases in May 2021. Studies assessing the association of GNRI with all-cause mortality or major adverse cardiovascular events (MACEs) in patients with CAD were included. The predictive value of GNRI was summarized by pooling multivariable adjusted risk ratios (RR) with 95% confidence intervals (CI) per GNRI point decrease or the lowest vs. the highest GNRI group.

**Results:** A total of eight studies involving 9277 patients with CAD were analyzed. Meta-analysis showed that the lowest GNRI was associated with a higher risk of all-cause mortality (RR 2.10; 95% CI 1.68–2.63) and MACEs (RR 2.84; 95% CI 1.56–5.16), respectively. Furthermore, per point decrease in GNRI was associated with 8 and 10% additional risk of all-cause mortality and MACEs. Subgroup analysis indicated that the value of low GNRI in predicting all-cause mortality was not affected by subtype of patients or follow-up duration.

**Conclusion:** Low GNRI score at baseline was associated with a higher risk of all-cause mortality and cardiovascular events in patients with CAD. The nutritional state estimated by the GNRI score could provide important predictive information in patients with CAD.

## Introduction

Geriatric Nutritional Risk Index, created by Bouillanne et al., was calculated by serum albumin level and the ratio of actual to ideal body weight (1). This new nutritional tool is designed to predict the risk of morbidity and survival in hospitalized elderly patients who find it difficult to obtain the normal weight. Under this simple nutritional tool, low GNRI score reflects poor nutritional status (2). Thereafter, this nutritional tool has been widely applied to evaluate the association of nutritional status and its adverse outcomes in various populations.

Despite the prevalence of nutritional deficiency remaining unclear in patients with coronary artery disease (CAD), malnutrition was reported to be at 38.7% with Controlling Nutritional status (CONUT) scores and 64% with GNRI scores among non-ST-elevated myocardial infarction (NSTEMI) (3). Poor nutritional status in patients with CAD is associated with unfavorable outcomes. Increasing pieces of evidence have suggested that malnutrition, estimated by low GNRI, was associated with an increased risk of mortality in patients with heart failure (4), hemodialysis (5), and various malignancies (6). Several studies (7–11) have investigated the associations of GNRI with adverse outcomes in patients with CAD. However, the predictive value of GNRI was not fully established in this population due to the presence of conflicting results (3, 12).

There is no previous meta-analysis specifically focused on the predictive value of GNRI in patients with CAD. We hypothesized that low GNRI scores may be associated with adverse outcomes. To summarize the available evidence, we performed this meta-analysis to assess the value of baseline GNRI in predicting adverse outcomes in patients with CAD in terms of all-cause mortality and major adverse cardiovascular events (MACEs).

## Methods

### Data Sources and Literature Searches

This study was reported according to the guideline of the Preferred Reporting Items for Systematic Reviews and Meta-Analyses (PRISMA) (13). Relevant studies were identified by comprehensively searching PubMed and Embase databases through May 2021. The following keywords in combination were applied for the literature search: “geriatric nutritional risk index” OR “GNRI” AND “coronary artery disease” OR “coronary heart disease” OR “acute coronary syndrome” OR “myocardial infarction” OR “unstable angina pectoris” (Supplementary Text 1). In addition, we also reviewed the reference lists of pertinent articles to identify any possible missing studies.

### Inclusion and Exclusion Criteria

Studies satisfying all the following criteria were included: (1) cohort studies recruiting patients with stable or acute CAD, (2) assessing the association of GNRI with all-cause mortality or major adverse cardiovascular events [(MACEs) including death, revascularization, no-fatal myocardial infarction, stroke, heart failure, or cerebrovascular attack], and (3) providing multivariable adjusted risk ratio (RR), hazard ratio (HR), or odds ratio (OR) with 95% confidence intervals (CI) per GNRI point decrease or the lowest vs. the highest GNRI group.

Studies reporting in-hospital outcomes were excluded.

### Data Extraction and Quality Assessment

The following information was collected from the included studies: the surname of the first author, published year, region, study design, subtypes of patients, sample sizes, percentage of men, age at baseline, cutoff value of the highest GNRI, definition of MACEs, time of follow-up, endpoints, most comprehensively adjusted risk summary, and adjusted confounders. The methodological quality of included studies was judged using the Newcastle–Ottawa Scale (NOS) (14). Studies with overall NOS ≥7 points were deemed to have high-quality. The above procedures were performed by two independent authors and disagreements were settled through discussion.

### Statistical Analysis

The STATA 12.0 (Stata Corporation, College Station, TX, USA) was applied to perform the meta-analysis. The predictive value of GNRI was calculated by pooling multivariable adjusted RR with 95% CI per point decrease in GNRI or the lowest vs. the highest category of GNRI. The *I*^2^ statistic and the Cochrane Q test were used to judge the heterogeneity, with statistical significance set at *I*^2^ ≥ 50% or *p* <0.10. We chose a random effects model in case of significant heterogeneity. Otherwise, a fixed-effect model was selected. To investigate the robustness of the pooling risk estimate, we conducted the sensitivity analysis by sequentially removing each study to recalculate the risk summary. Meanwhile, we performed the subgroup analyses according to the subtypes of patients with CAD, sample sizes, median or mean age, and length of follow-up. Both Begg's test (15) and Egger's test (16) were planned to examine the likelihood of publication bias when the outcomes included more than 10 studies.

## Results

### Search Results and Study Characteristics

**Figure 1:**
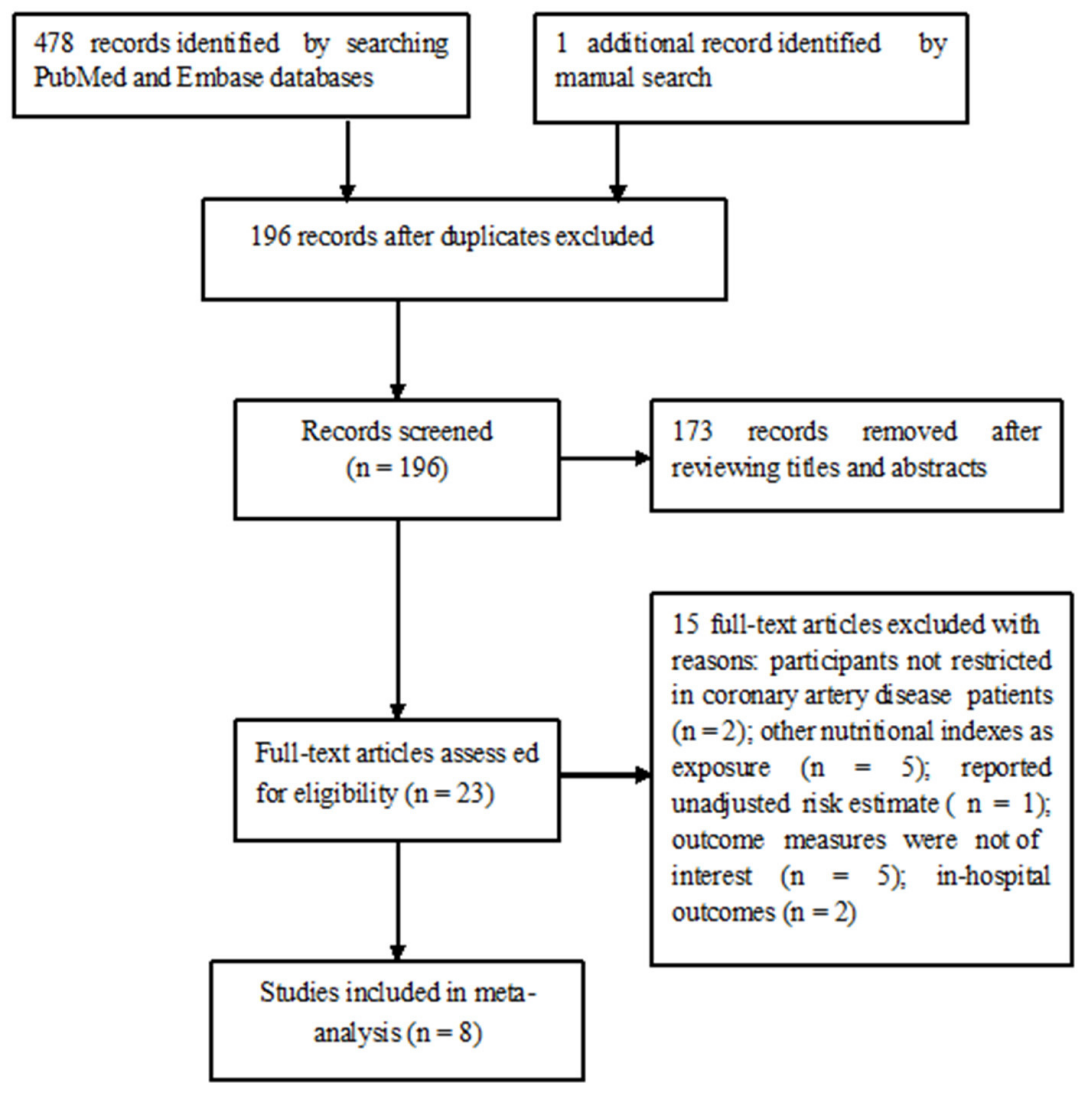
Flowchart showing the study selection process.

Figure 1 summarizes the process of study selection. A total of eight studies (7–12, 17, 18) involving 9,277 patients with CAD were included in this meta-analysis. The main characteristics of these eligible studies are summarized in Table 1. The included studies were published from 2016 to 2021, with sample sizes ranging between 206 and 2853. All the included studies were performed in Asian countries (Republic of Korea, Japan, and China). Five studies (7–9, 11, 18) were based on patients with total CAD and three studies (10, 12, 17) were based on the acute stage of CAD. The median or mean age of the patients ranged from 60 to 74 years. The time of follow-up ranged between 1 and 7.4 years. Regarding the methodological quality, the included studies were grouped as high quality (Supplementary Table 1).

**Table 1:** Main characteristics of the included studies.

**References**	**Country**	**Study design**	**Patients (% men)**	**Age (years)**	**GNRI cutoff value**	**Definition of MACEs**	**Follow-up (years)**	**Outcomes/HR (95% CI)**	**Adjusted variables**	**Total NOS**
Huang et al. (7)	China	P	CAD 1772 (73)	72.5 ± 5	<98 vs. ≥98	—	27 months	Total death 1.99 (1.35–2.95)	Age, sex, clinical diagnosis, SBP, creatinine, hemoglobin, DM, LVEF, coronary disease, usage of drugs, PCI, hypertension	8
Kunimura et al. (8)	Japan	P	CAD 802 (69)	70 ± 10	<92 vs. >98	Cardiac death or non-fatal MI	4.3 years	MACEs 6.76 (3.13–14.56)	Age, sex, current smoker, DM, hypertension, dyslipidemia, eGFR, statins, BNP	8
Wada et al. (9)	Japan	R	CAD 2853 (82.1)	65.9 ± 10.3	<98 vs. >104; Per point decrease	—	7.4 years	Total death 1.87 (1.31–2.71) 1.04 (1.03–1.06)	Age, chronic kidney disease, DM, hs-CRP LVEF, multivessel disease, use of statins	8
Zhou et al. (10)	China	R	STEMI 309 (80.9)	58.4 ± 12.9	<94 vs. ≥94	—	19.5 months	Total death 2.04 (1.04–4.00)	Age, lactate dehydrogenase, urea nitrogen, creatinine, triglyceride, hemoglobin, red blood cell distribution width, WBC, LVEF, troponin T, Killip class, GRADE score	8
Katayama et al. (11)	Japan	R	CAD 206 (63.1)	74 (67–81)	Per point decrease	Death, MI, revascularization	12 months	MACEs 1.06 (1.03–1.09)	Age, sex, BMI, albumin, hemodialysis, total lymphocytes, history of PCI	7
Zhao et al. (12)	China	R	ACS 1519 (72.2)	60 ± 8.91	<103.6 vs. ≥103.6; Per point decrease	Death, non-fatal MI, revascularization	12 months	Total death 5.26 (0.52–53.4) 1.32 (1.11–1.58) MACEs 2.41 (1.80–3.22) 1.16 (1.13–1.19)	Age, prior MI or PCI, triglyceride, hs-CRP, left-main disease, chronic total occlusion, bifurcation lesion, number of stents	7
Kim et al. (17)	Republic of Korea	R	AMI 1147 (72.5)	65.6 (64.9–66.4)	<112.3 vs. >112.3; Per point decrease	Cardiac death, re-AMI, HF, CVA, revascularization	12 months	Total death 3.01 (1.26–7.16) 1.08 (1.04–1.12) MACEs 1.08 (1.04–1.10)	Age, sex, current smoker, creatinine, hypertension, DM, BMI, LVEF, multivessel disease, baseline diagnosis	7
Cheng et al. (18)	China	R	CAD 669 (83.6)	65.32 ± 9.97	<92 vs. ≥98	Death, non-fatal MI, revascularization, stroke	33 months	Total death 2.90 (1.43–5.87) MACEs 1.76 (1.02–3.03)	Age, sex, smoking, alcohol, hypertension, DM, TC, sent numbers, LVEF, BNP, creatinine, hs-CRP, heart rate, revascularization, clinical frailty scale	8

*GNRI, geriatric Nutritional Risk Index; RR, risk ratio; HR, hazard ratio; CI, confidence intervals; R, retrospective; P, prospective; BMI, body mass index; BNP, brain natriuretic peptide; HF, heart failure; hs-CRP, high-sensitivity C-reactive protein; TC, total cholesterol; DM, diabetes mellitus; LVEF, left ventricular ejection fraction; CVA, cerebrovascular attack; MI, myocardial infarction; eGFR, estimated glomerular filtration rate; GRADE, Grading of Recommendations, Assessment, Development and Evaluation; NOS, Newcastle-Ottawa Scale*.

### All-Cause Mortality

**Figure 2:**
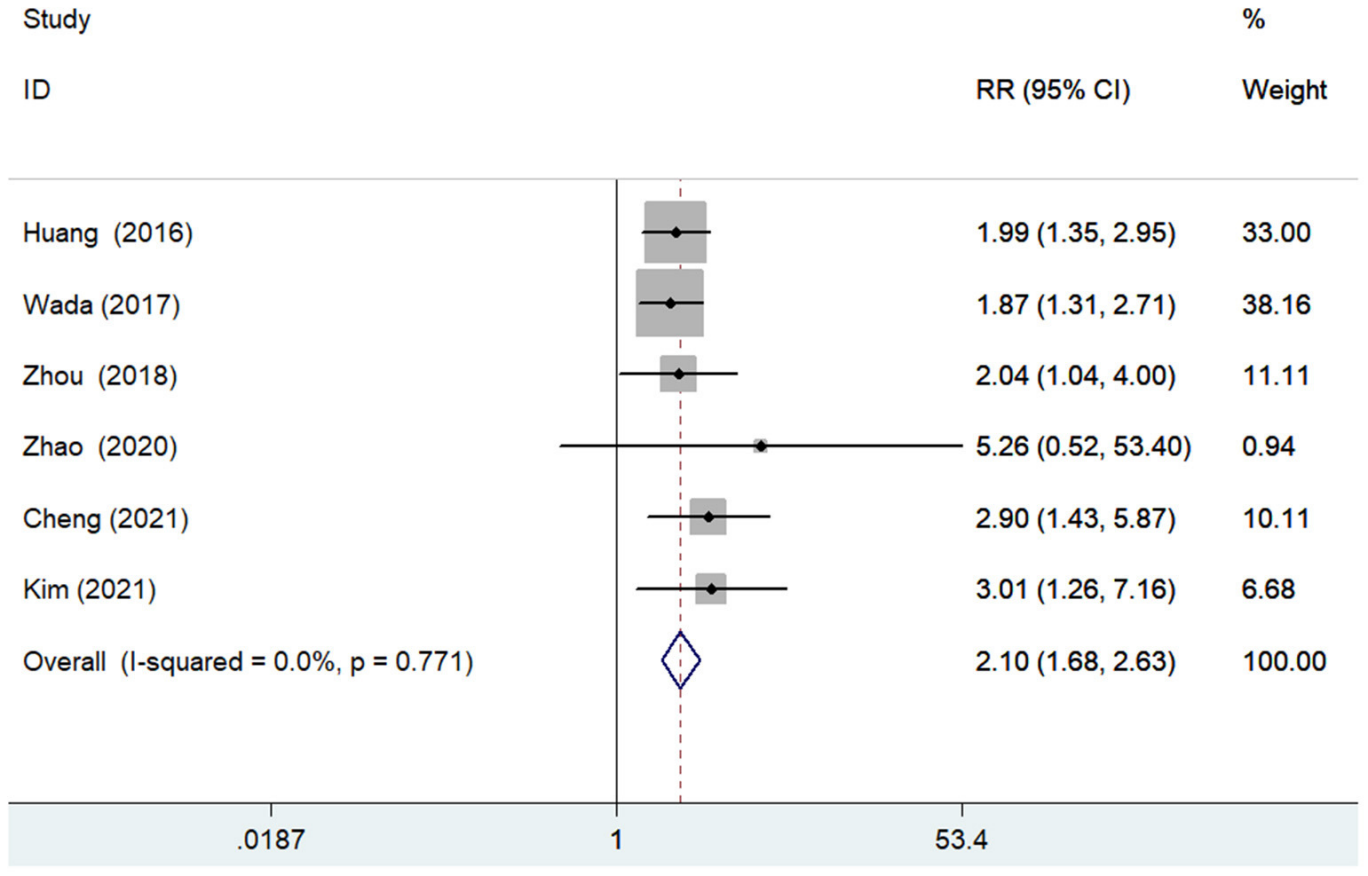
Forest plots showing the pooled RR and 95% CI of all-cause mortality for the lowest vs. the highest GNRI category. RR, risk ratio; CI, confidence interval; GNRI, Geriatric Nutritional Risk Index.

**Figure 3:**
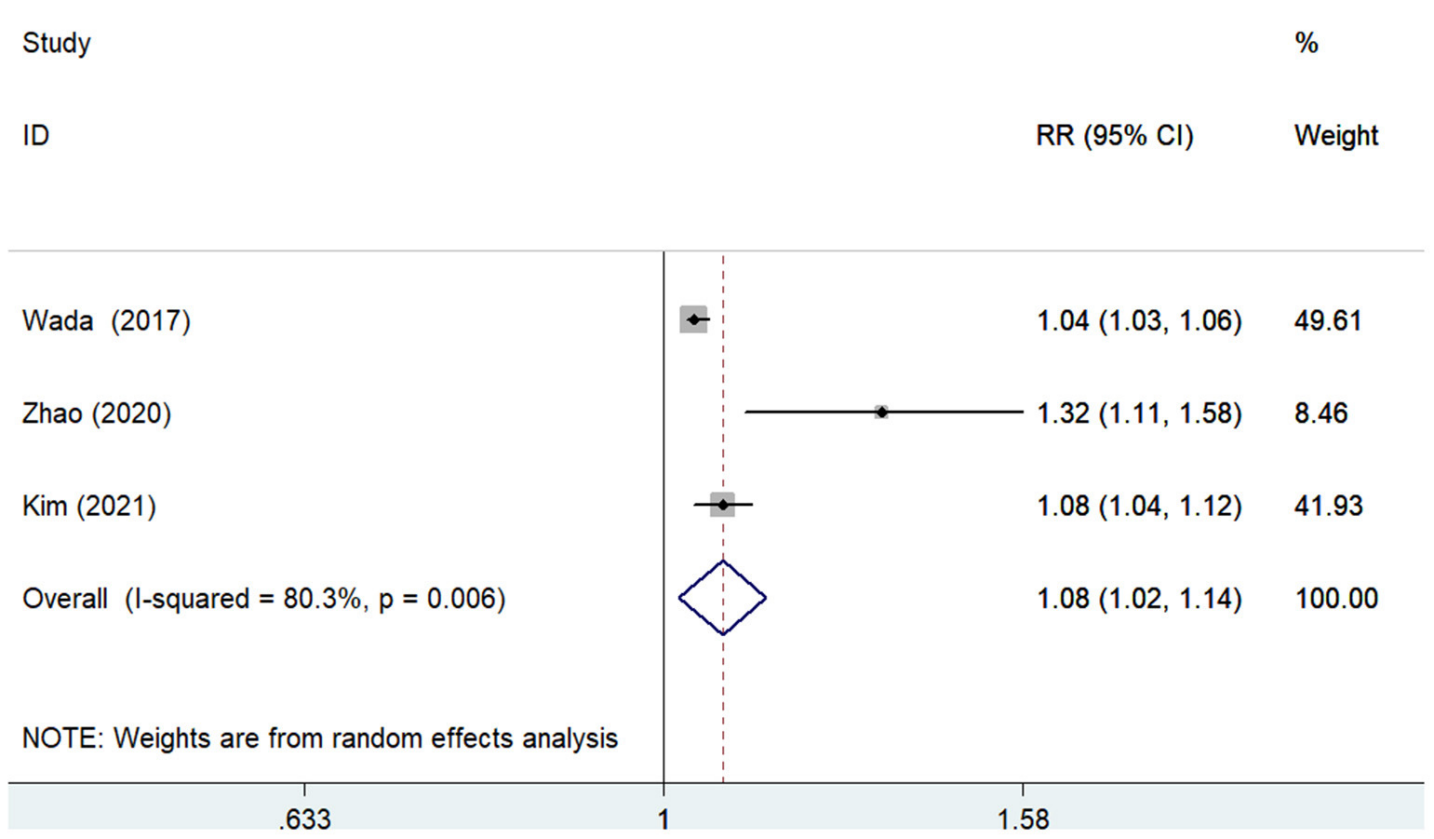
Forest plots showing the pooled RR and 95% CI of all-cause mortality for per point decrease in GNRI. RR, risk ratio; CI, confidence interval; GNRI, Geriatric Nutritional Risk Index.

The predictive value of GNRI by categorical analysis for all-cause mortality was available in six studies (7, 9, 10, 12, 17, 18). As shown in Figure 2, there was no evidence of significant heterogeneity (*I*^2^ = 0%; *p* =0.771). The adjusted pooled RR of all-cause mortality was 2.10 (95% CI 1.68–2.63) when compared the lowest to the highest GNRI category. The value of the lowest GNRI in predicting all-cause mortality was consistently observed across multiple subgroups (Supplementary Table 1). In addition, three studies (9, 12, 17) assessed the predictive value of GNRI by continuous data for all-cause mortality. As shown in Figure 3, the pooled RR of all-cause mortality was 1.08 (95% CI 1.02–1.14) per point decrease in GNRI, with significant heterogeneity (*I*^2^ = 80.3%; *p* = 0.006). Sensitivity analysis indicated that removal of individual study each time did not significantly alter the originally statistical significance of the pooling risk estimate (data not shown).

### Major Adverse Cardiovascular Events

**Figure 4:**
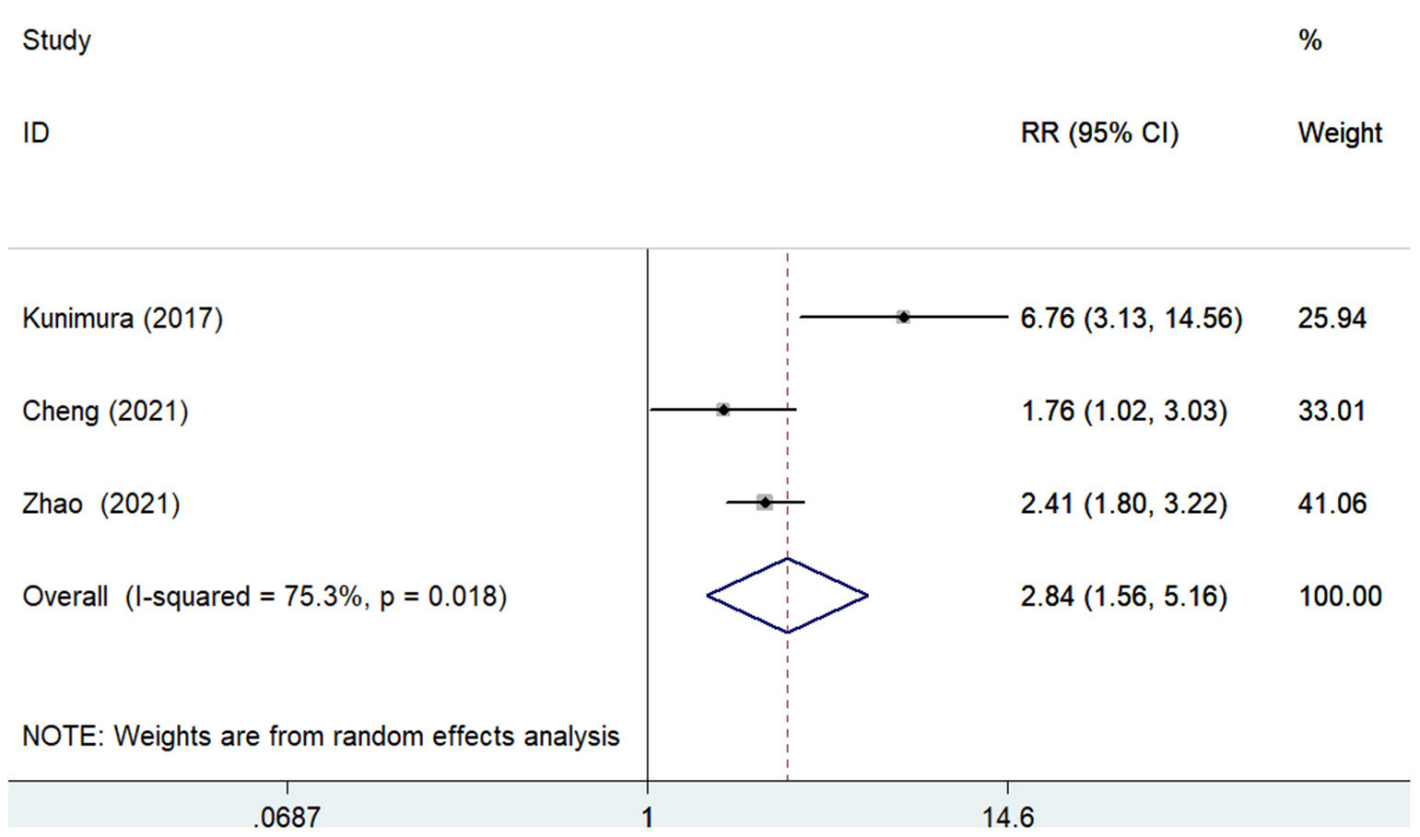
Forest plots showing the pooled RR and 95% CI of major adverse cardiovascular events for the lowest vs. the highest GNRI category. RR, risk ratio; CI, confidence interval; GNRI, Geriatric Nutritional Risk Index.

**Figure 5:**
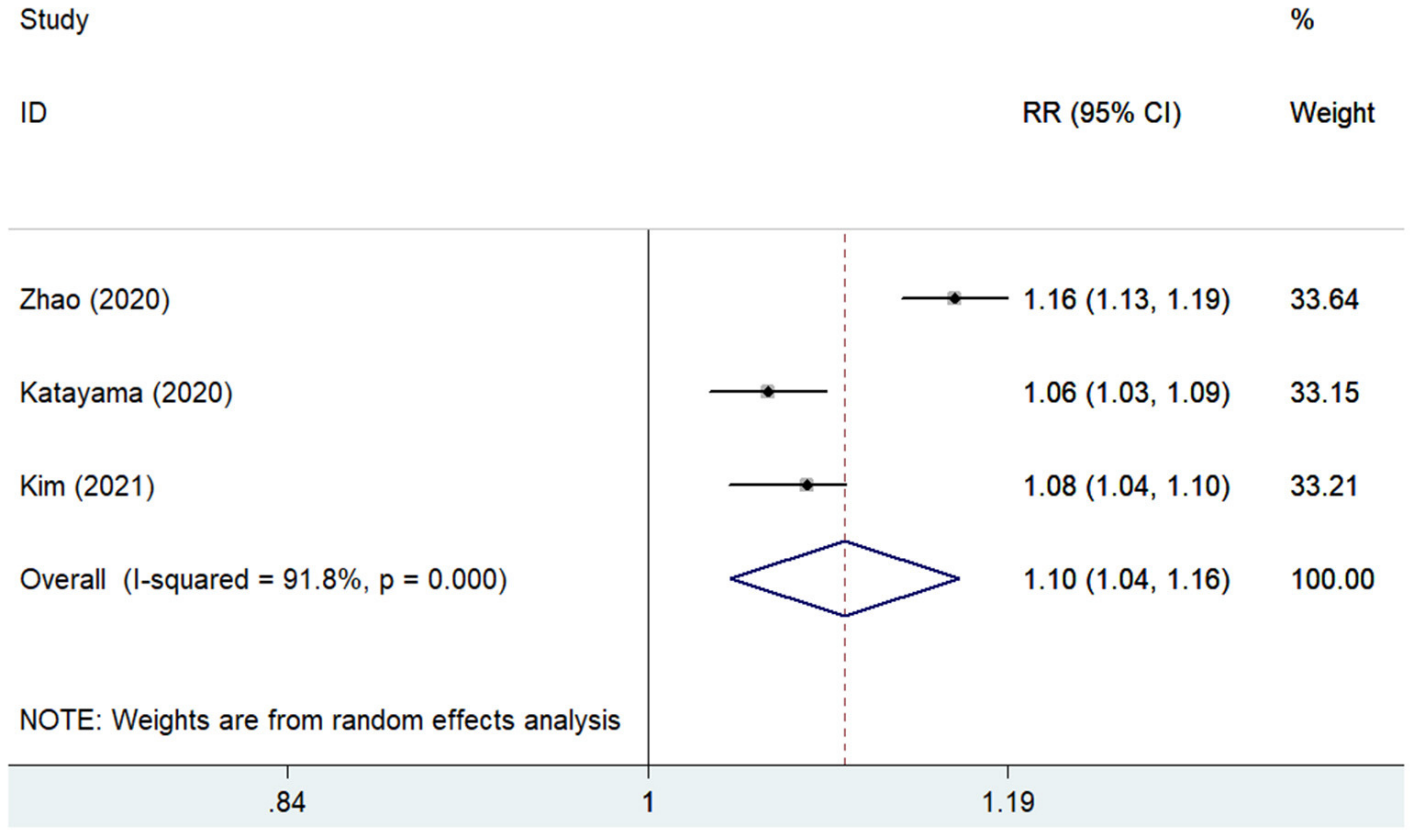
Forest plots showing the pooled RR and 95% CI of major adverse cardiovascular events for per point decrease in GNRI. RR, risk ratio; CI, confidence interval; GNRI, Geriatric Nutritional Risk Index.

The predictive value of GNRI for MACEs by categorical analysis was available in three studies (8, 12, 18). As shown in Figure 4, there was evidence of significant heterogeneity (*I*^2^ = 75.3%; *p* = 0.018). The adjusted pooled RR of MACEs was 2.84 (95% CI 1.56–5.16) when compared the lowest to the highest GNRI category. In addition, three studies (11, 12, 17) evaluated the value of GNRI by continuous data in predicting MACEs. As shown in Figure 5, the pooled RR of MACEs was 1.10 (95% CI 1.04–1.16) per point decrease in GNRI, with significant heterogeneity (*I*^2^ = 91.8%; *p* < 0.001). Sensitivity analysis suggested that the originally statistical significance of the pooling risk estimate did not significantly change when excluded from the individual study each time (data not shown).

### Publication Bias

We did not conduct the Begg's test and the Egger's test to check the publication bias because of the less than recommended arbitrary number of 10 studies (19).

## Discussion

This is the first meta-analysis to evaluate the predictive value of GNRI in patients with CAD. The main findings of our meta-analysis indicated that a low GNRI score was an independent predictor risk all-cause mortality and MACEs in patients with CAD. The patients with CAD who had the lowest GNRI score had a 2.84-fold and 2.10-fold increased risk of MACEs and all-cause mortality, respectively. Moreover, per point decrease in GNRI score was associated with 10 and 8% higher risk of MACEs and all-cause mortality, respectively. Together with these findings, the nutritional status, estimated by the GNRI, may provide important predictive value in patients with CAD.

Geriatric Nutritional Risk Index score was also associated with a 3.16-fold higher risk of bleeding event in patients undergoing percutaneous coronary intervention (PCI) with an oral anticoagulant during a 3-year follow-up (20). In addition, each 1-point decrease in GNRI score significantly increased 6.5% higher risk of cardiovascular mortality in patients with ST-segment elevation myocardial infarction (STEMI) during the 12.4 month follow-up period (21). Apart from the long-term outcomes, GNRI (<92) at admission was an independent factor influencing post-myocardial infarction complications (OR 2.13; 95%CI 1.61–2.84) and in-hospital death (OR 2.48; 95%CI 1.55–3.95) in patients with acute myocardial infarction (22). The findings above further supported the predictive role of GNRI in patients with CAD.

Several nutritional scoring systems, including the prognostic nutritional index (PNI), CONUT, triglycerides-total cholesterol-body weight index (TCBI), Mini Nutritional Assessment (MSA), Graz Malnutrition Screening (GMS), and GNRI have been introduced for evaluating nutritional state in clinical practice. Malnutrition determined by the PNI (23), TCBI (24, 25), and CONUT (3) was also significantly associated with adverse outcomes in patients with CAD (23). However, there is no consensus on which tools have the best predictive role in patients with CAD. GNRI is a simple tool for assessing the nutritional status of the aging population (1). Considering that the majority of included patients with CAD were from the elderly population, GNRI may be the best tool for evaluating nutritional status in these patients. Therefore, our meta-analysis only focused on the predictive role of GNRI in patients with CAD. Among patients with acute myocardial infarction, GNRI was reported to have the best value in predicting all-cause mortality than the TCBI and PNI scoring systems (17). Using the area under the curve analysis, the GNRI score had a stronger predictive value for cardiovascular death than those of the PNI and CONUT score (21). Our future study will further evaluate the predictive value of malnutrition defined by other nutritional tools in patients with CAD or compare which tool has the best predictive value. It should be noted that combined, the different scoring systems could achieve the greatest incremental value in predicting adverse cardiovascular outcomes (26).

Coronary artery disease includes a heterogeneous population. Results of subgroup analysis suggested that the association between low GNRI and all-cause mortality was stronger in patients with acute coronary syndrome (ACS) compared with those with stable CAD. The value of low GNRI in predicting all-cause mortality weakened with the lengthening of the follow-up time in the subgroup analysis. It is noteworthy that these results were established on the small number of studies analyzed. Future studies are necessary to confirm the present findings.

Despite diet nutrition being recommended in patients with CAD as secondary prevention, nutritional support is often neglected by physicians (27). This meta-analysis underlines the importance to evaluate the nutritional state in patients with CAD. Nutritional deficiency estimated by the GNRI may provide important predictive information in patients with CAD regardless of the stage (acute or stable). Malnutrition is a modifiable risk factor. CAD patients with malnutrition should be given closer monitoring, dietary intervention, and intensive treatment. However, whether nutritional intervention can improve the prognosis of CAD patients with malnutrition has not been demonstrated in clinical trials.

There are several potential limitations in the current meta-analysis. First, the majority of the analyzed studies adopted the retrospective design, and selection bias of this type of study may have been committed. Second, the included studies selected different thresholds of low GNRI scores, which makes it difficult for clinicians to identify those in need of nutritional supplementation. Third, there was significant heterogeneity in pooling MACE subtypes. Different subtypes of patients with CAD, thresholds of low GNRI score, definitions of MACEs, or intervals of follow-up duration may be correlated to the observed heterogeneity. Fourth, GNRI score of 92–98, 82–91, and <82 reflects the mild, moderate, and severe malnutrition, respectively. However, most of the included studies reported the predictive value of GNRI based on the single cutoff and not by nutritional status, which prevents the evaluation of the prediction of malnutrition defined by GNRI <92. Future studies should further assess the association of the different degrees of malnutrition with adverse outcomes in patients with CAD. Fifth, considering all patients were from East Asia, generalization of the current findings to western countries should be done with caution. Finally, we did not examine the publication bias tests due to the less than recommended arbitrary minimum number of studies.

## Conclusions

This meta-analysis consolidates the growing evidence that a lower GNRI score at baseline is an independent predictor of all-cause mortality and MACEs in patients with CAD regardless of the acute stage of stable phase. Nutritional status estimated by the GNRI score may play an important role in the risk classification of patients with CAD.

## Data Availability Statement

The original contributions presented in the study are included in the article/Supplementary Material, further inquiries can be directed to the corresponding author.

## Author Contributions

CFM: study conception, design, and revising the article critically for important intellectual content. YF and LH: acquisition of data, analysis, and interpretation of data. YF and YJZ: statistical analysis. YF: drafting the article. All authors read and approved the final version of the study to be published.

## Funding

This study was supported by (1) Jiangsu Innovative Team Leading Talent Fund (CXTDC2016006, QNRC2016446), (2) Jiangsu 333 Talent Fund (BRA2020016), and (3) Suqian Science and Technology Support Project Fund (K201907).

## Conflict of Interest

The authors declare that the research was conducted in the absence of any commercial or financial relationships that could be construed as a potential conflict of interest.

## Publisher's Note

All claims expressed in this article are solely those of the authors and do not necessarily represent those of their affiliated organizations, or those of the publisher, the editors and the reviewers. Any product that may be evaluated in this article, or claim that may be made by its manufacturer, is not guaranteed or endorsed by the publisher.
