# Supplementary material for: Predictive Value of Geriatric Nutritional Risk Index in Patients With Coronary Artery Disease: A Meta-Analysis
Source: Front Nutr. 2021 Sep 29;8:736884. doi: 10.3389/fnut.2021.736884 (PMC8511313; doi:10.3389/fnut.2021.736884)
Supplement: Supplementary file 1 [file Data_Sheet_1.pdf]

Supplemental Text S1 – Search strategy for this meta-analysis

Medical Databases---PubMed and Embase

Limits: English and Chinese language, publications until May 31,2021

1 (“coronary artery disease” [MeSH Terms] OR “coronary heart disease” [MeSH Terms] OR “acute coronary syndrome” [MeSH Terms] OR “myocardial infarction” [MeSH Terms] OR “unstable angina pectoris” [MeSH Terms])

2 (“geriatric nutritional risk index” [MeSH Terms] OR “GNRI” [Free Terms])

3 (#1) AND (#2)

In addition, reference lists of the analyzed studies and pertinent articles were manually scanned.
