# Supplementary material for: Predictive Value of Geriatric Nutritional Risk Index in Patients With Coronary Artery Disease: A Meta-Analysis
Source: Front Nutr. 2021 Sep 29;8:736884. doi: 10.3389/fnut.2021.736884 (PMC8511313; doi:10.3389/fnut.2021.736884)
Supplement: Supplementary file 2 [file Table_1.pdf]

Supplemental Table S1 Methodological quality assessment based on the Newcastle-Ottawa Scale

Author/Year	Representativeness of the exposed cohort	Selection of the non-exposed cohort	Ascertainment of exposure	Demonstration that outcome was not present at study start	Comparability of cohorts on the basis of the design or analysis	Assessment of outcome	Enough follow-up periods (> 12 months)	Adequacy of follow-up of cohorts	Total scores
Huang 2016 (7)	★	★	★	★	★	★	★	★	8
Kunimura 2017 (8)	★	★	★	★	★	★	★	★	8
Wada 2017 (9)	★	★	★	★	★	★	★	★	8
Zhou 2018 (10)		★	★	★	★★	★	★	★	8
Katayama 2020 (11)	★	★	★	★	★	★		★	7
Zhao 2020 (12)		★	★	★	★★	★		★	7
Kim 2021 (17)		★	★	★	★★	★		★	7
Cheng 2021 (18)	★	★	★	★	★	★	★	★	8
